# Active components from Radix Scrophulariae inhibits the ventricular remodeling induced by hypertension in rats

**DOI:** 10.1186/s40064-016-1985-z

**Published:** 2016-03-22

**Authors:** Chao Chao Zhang, Wei Liang Gu, Xi Min Wu, Yi Ming Li, Chang Xun Chen, Xiao Yan Huang

**Affiliations:** Experiment Center for Science and Technology, Shanghai University of Traditional Chinese Medicine, 1200 Cailun Road, Shanghai, 201203 People’s Republic of China; Department of Pharmacology, Shanghai University of Traditional Chinese Medicine, 1200 Cailun Road, Shanghai, 201203 People’s Republic of China; Department of Natural Product Chemistry, Shanghai University of Traditional Chinese Medicine, 1200 Cailun Road, Shanghai, 201203 People’s Republic of China

**Keywords:** Radix Scrophulariae, Active component, Hypertension, Ventricular remodeling

## Abstract

**Background:**

In the previous study, active extract of Radix Scrophularia (ACRS) demonstrated beneficial effects on ventricular remodeling induced by coronary artery ligation and lowered blood pressure in rats. And ACRS also exhibited the effect on lowering the blood pressure in spontaneously hypertensive rats (SHRs). The aim of this study is to explore the effects of ACRS on ventricular remodeling in SHRs and underlying mechanisms.

**Results:**

ACRS significantly lowered the blood pressure, decreased the heart mass indexes, inhibited the deposition of perivascular and interstitial, attenuated the accumulation of types I and III collagen, reduced the tissue angiotensin II, serum norepinephrine and tumor necrosis factor-α concentrations. The underlying mechanisms may be related to downregulating the mRNA expressions of collagen type I, transforming growth factor-β1 and angiotensin converting enzyme, suppressing the phosphorylation of extracellular signal regulated kinase 1/2, c-Jun N-terminal kinase (JNK/SAPK) and p38 mitogen-activated protein kinases (p38 MAPK).

**Conclusion:**

Continuous treatment of SHRs with ACRS for 21 weeks reduced blood pressure, myocardial hypertrophy and the amount of interstitial and perivascular collagen, which indicated that ACRS could prevent hypertensive ventricular remodeling. This can be attributed to suppression of the sympathetic nervous and renin angiotensin aldosterone system through the inhibition of ERK 1/2, JNK and p38 MAPK pathways.

## Background

Heart failure (HF) is characterized by decreased cardiac function and associated with pathological left ventricular remodeling (Gerdes 2002). Based on population attributable risks, hypertension has the greatest impact, accounting for 39 % of HF events in men and 59 % in women (Kannel 2000). Hypertensive heart disease involves alterations in cardiac structure and function, including interstitial and perivascular fibrosis, leading eventually to impaired myocardial performance and coronary haemodynamics (Bartha et al. 2009).

Radix Scrophularia (Xuanshen) is a traditional Chinese herb medicine derived from the *Scrophularia ningpoensis* Hemsl, has been widely used in many prescriptions for treating cardiovarcular diseases, including hypertension and myocardial ischemia (Wagner et al. 2011). In the previous study, we revealed that active extract of Radix Scrophularia (ACRS) exhibited beneficial effects on ventricular remodeling in rats induced by coronary artery ligation (Huang et al. 2012). And ACRS also exhibited the effect on lowering the blood pressure (Chen et al. 2012). However, the influence of ACRS on ventricular remodeling induced by hypertension is not clear. In this paper, the effect of ACRS on ventricular remodeling in spontaneously hypertensive rat (SHR) is investigated.

## Results

### Blood pressure

**Fig. 1:**
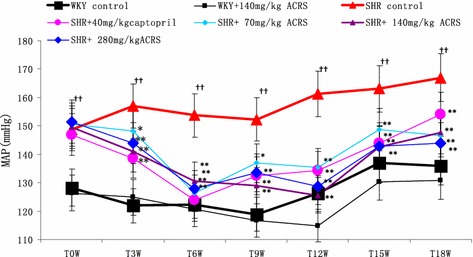
Effects of active components of Radix Scrophulariae (ACRS) on mean arterial pressure (MAP) of spontaneously hypertensive rats (SHR) and Wistar–Kyoto rats (WKY) ($\bar{x}$ ± SE, n = 8). Compared with WKY control group, ^††^ *P* < 0.01. Compared with SHR control group, *P < 0.05; **P < 0.01

As illustrated in Fig. 1, ACRS at 140 mg/kg had no adverse effects on mean arterial pressure (MAP) in normotensive age-matched Wistar–Kyoto rats (WKY). MAP was significantly elevated from ten weeks old (the beginning of treatment) in SHR model group but not in WKY rats (*P* < 0.05).

MAP declined as animals became older by chronic treatment with ACRS at doses of 70, 140 and 280 mg/kg each day, for 21 weeks (*P* < 0.05, *P* < 0.01), and also with captopril (*P* < 0.01).

### Cardiac mass index

The physical characteristics of each group are presented in Table 1, ACRS at 140 mg/kg had no adverse effects on left ventricular mass index (LVMI, mg/g) and heart mass index (HMI, mg/g) in normotensive age-matched WKY. However, the LVMI and HMI of SHR control were significantly greater than WKY control (*P* < 0.01). Treated with ACRS significantly lowed LVMI and HMI obviously, as well as captopril (*P* < 0.05, *P* < 0.01).

**Table 1:** Effects of active components of Radix Scrophulariae (ACRS) on cardiac mass index of spontaneously hypertensive rats (SHR) and Wistar–Kyoto rats (WKY) ($\bar{x}$ ± SE, n = 8)

Group	LVWI (mg/g)	HWI (mg/g)
WKY control	2.36 ± 0.15	3.02 ± 0.19
WKY+ 140 mg/kg ACRS	2.49 ± 0.43	3.28 ± 0.85
SHR control	3.09 ± 0.24^††^	3.75 ± 0.24^††^
SHR+ 40 mg/kg captopril	2.50 ± 0.09**	3.15 ± 0.13**
SHR+ 70 mg/kg ACRS	2.80 ± 0.11**	3.35 ± 0.11**
SHR+ 140 mg/kg ACRS	2.90 ± 0.16*	3.46 ± 0.20**
SHR+ 280 mg/kg ACRS	2.87 ± 0.14**	3.45 ± 0.16**

Compared with WKY control group: ^††^ *P* < 0.01; compared with SHR control group: * *P* < 0.05; ** *P* < 0.01

*LVMI* left ventricular mass index (mg/g), *HMI* heart mass index (mg/g)

### Collagen accumulation

**Fig. 2:**
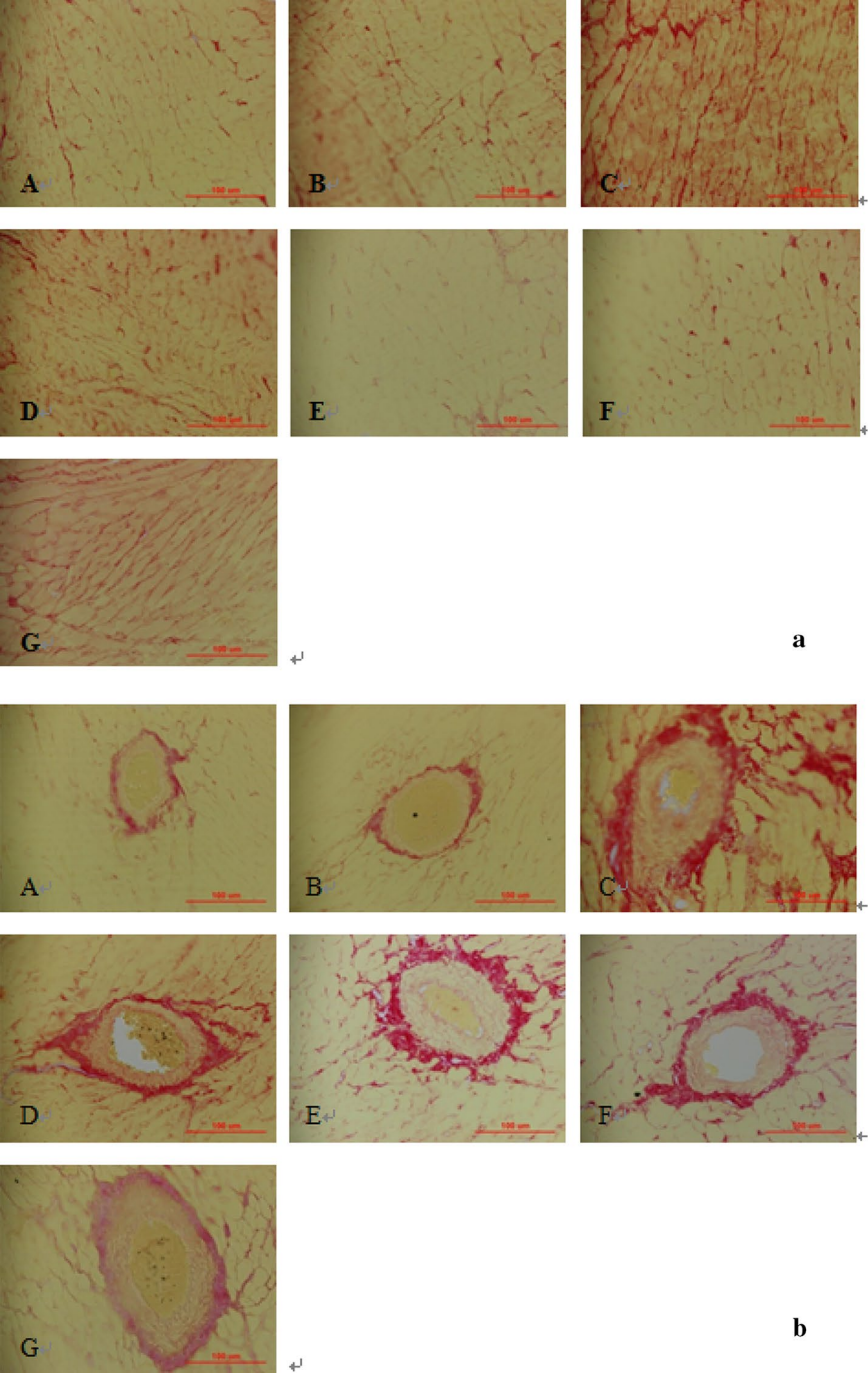
Effects of active components of Radix Scrophulariae (ACRS) on cardiac collagen in the interstitial (**a**) and perivascular (**b**) space of the left ventricle in spontaneously hypertensive rats (SHR) and Wistar–Kyoto rats (WKY) (Sirius red stain, ×400). **A** WKY control; **B** WKY+ 140 mg/kg ACRS; **C** SHR control; **D** SHR+ 40 mg/kg captopril; **E** SHR+ 70 mg/kg ACRS; **F** SHR+ 140 mg/kg ACRS; **G** SHR+ 280 mg/kg ACRS

As illustrated in Fig. 2, the collagen showed red or deep red in myocardial interstitial and perivascular, pink or orange was myocytes. There was little interstitial and perivascular collagen in WKY rats with or without ACRS. There was a large amount of interstitial and perivascular collagen appeared in SHR model. Collagen deposition in SHR with ACRS or captopril was less than that in SHR model (Table 2).

**Table 2:** Effects of active components of *Radix Scrophulariae* (ACRS) on interstitial collagen volume fraction (ICVF), perivascular collagen area to luminal area ratio (PVCA), collagen types I and III volume fraction of the left ventricle in spontaneously hypertensive rats (SHR) and Wistar–Kyoto rats (WKY) ($\bar{x}$ ± SE, n = 6)

Group	ICVF (%)	PVCA (ratio)	Collage I (%)	Collage III (%)	I/III
WKY control	6.53 ± 4.10	0.78 ± 0.33	0.22 ± 0.20	0.06 ± 0.04	3.56 ± 2.24
WKY+ 140 mg/kg ACRS	4.29 ± 2.71	1.01 ± 0.66	0.26 ± 0.20	0.11 ± 0.07	2.89 ± 2.30
SHR control	22.55 ± 5.88^††^	10.36 ± 4.38^††^	7.78 ± 2.06^††^	0.49 ± 0.44^†^	33.58 ± 26.93^†^
SHR+ 40 mg/kg captopril	5.72 ± 4.08**	0.92 ± 0.37**	0.15 ± 0.10**	0.11 ± 0.05	2.76 ± 3.90*
SHR+ 70 mg/kg ACRS	7.51 ± 3.35**	1.59 ± 0.52**	0.42 ± 0.31**	0.13 ± 0.04	3.97 ± 4.18*
SHR+ 140 mg/kg ACRS	6.54 ± 2.08**	0.76 ± 0.42**	0.38 ± 0.31**	0.08 ± 0.05*	6.54 ± 7.79*
SHR+ 280 mg/kg ACRS	10.23 ± 7.20**	1.21 ± 0.75**	0.48 ± 0.65**	0.07 ± 0.06*	7.38 ± 10.37*

Compared with WKY control group: ^††^ *P* < 0.01; compared with SHR control group: * *P* < 0.05; ** *P* < 0.01

**Fig. 3:**
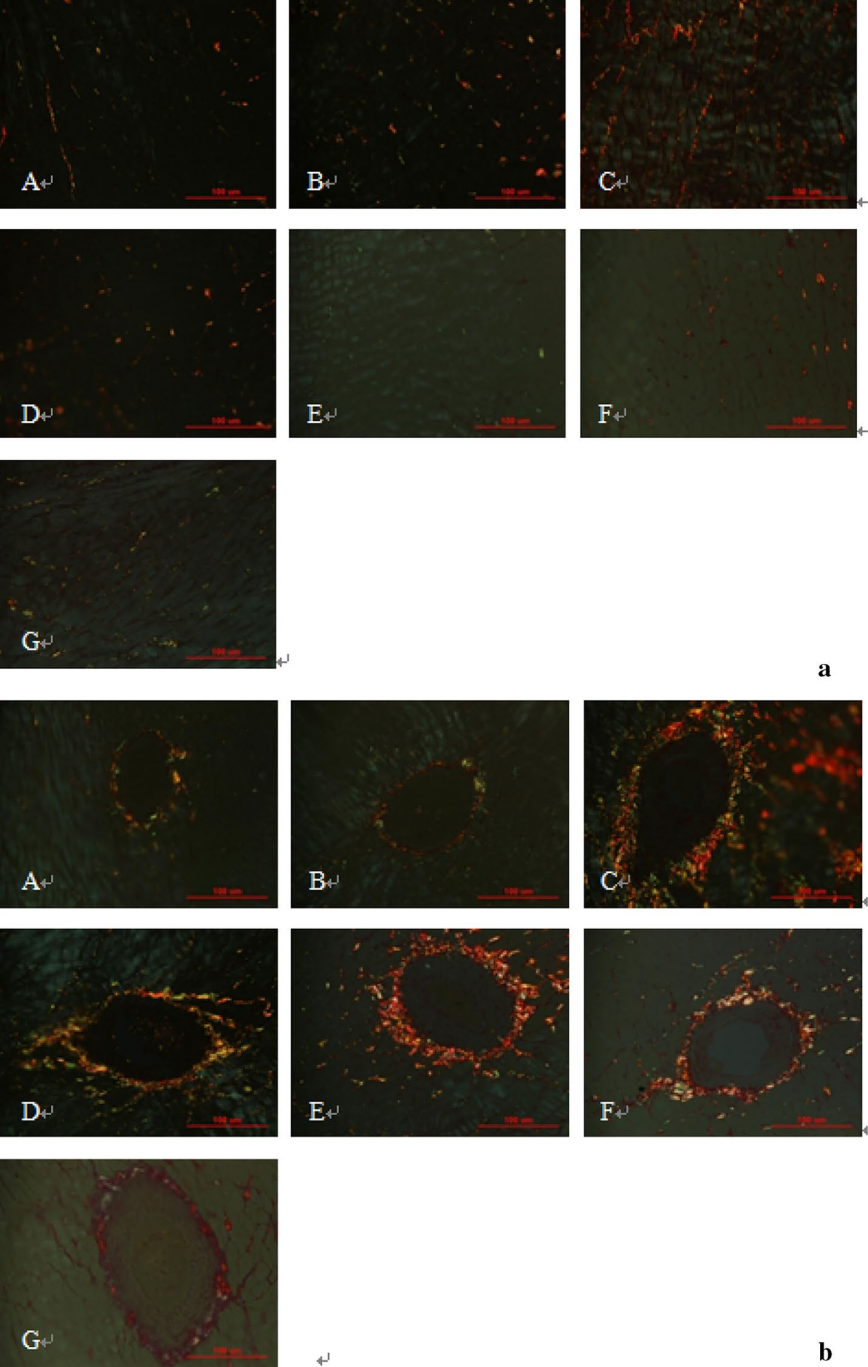
Effects of active components of Radix Scrophulariae (ACRS) on collagen type I, type III in the interstitial (**a**) and perivascular (**b**) space of the left ventricle in spontaneously hypertensive rats (SHR) and Wistar–Kyoto rats (WKY) (Sirius red stain and polarized light, ×400). **A** WKY control; **B** WKY+ 140 mg/kg ACRS; **C** SHR control; **D** SHR+ 40 mg/kg captopril; **E** SHR+ 70 mg/kg ACRS; **F** SHR+ 140 mg/kg ACRS; **G** SHR+ 280 mg/kg ACRS

Under the polarized light microscope, collagen of type III appeared red or yellow, collagen of type III appeared green. There was little accumulation of types I and III collagen in WKY rats with or without ACRS. The collagen I and III distribution of SHRs was significantly increased compared with WKY rats. ACRS or captopril reduced it significantly (Table 2; Fig. 3).

There was no change of the ratio of collagen type I/III in WKY with or without ACRS (Table 2). Higher ratio of collagen type I/III was found in SHR model than compared to WKY (*P* < 0.01). ACRS or captopril lowed it remarkably (*P* < 0.01).

### Serum norepinephrine (NE) concentration

As depicted in Table 3, significant difference of NE concentration was not found in WKY with or without ACRS. The protein level of serum NE was significantly increased in SHR model than WKY rats (*P* < 0.05). Treated with ACRS (140, 280 mg/kg) or captopril significantly lowed the protein level of serum NE (*P* < 0.05).

**Table 3:** Effects of active components of Radix Scrophulariae (ACRS) on serum concentration of norepinephrine (NE), myocardium Angiotensin II and serum level of tumor necrosis factor α (TNF-α) concentration in spontaneously hypertensive rats (SHR) and Wistar–Kyoto rats (WKY) ($\bar{x}$  ± SE, n = 6)

Group	NE (ng/ml)	Ang II (pg/mg·prot)	TNF-α (ng/L)
WKY control	0.15 ± 0.03	89.61 ± 29.51	24.95 ± 1.26
WKY+ 140 mg/kg ACRS	0.14 ± 0.01	62.75 ± 13.56	24.53 ± 0.92
SHR control	0.36 ± 0.20^†^	195.23 ± 113.35^†^	27.63 ± 1.96^†^
SHR+ 40 mg/kg captopril	0.16 ± 0.02*	91.07 ± 8.28*	24.11 ± 1.11*
SHR+ 70 mg/kg ACRS	0.37 ± 0.15	94.43 ± 58.96	22.89 ± 2.33**
SHR+ 140 mg/kg ACRS	0.15 ± 0.02*	71.86 ± 14.41*	24.08 ± 2.36*
SHR+ 280 mg/kg ACRS	0.18 ± 0.05*	72.79 ± 20.81*	23.04 ± 3.69*

Compared with WKY control group: ^†^ *P* < 0.05; compared with SHR control group: * *P* < 0.05; ** *P* < 0.01

### Serum tumor necrosis factor α (TNF-α) concentration

As depicted in Table 3, significant difference of TNF-α concentration was not found in WKY with or without ACRS. The protein level of serum TNF-α was significantly increased in SHR model than WKY rats (*P* < 0.05). Treated with ACRS (140, 280 mg/kg) or captopril significantly lowed the protein level of serum TNF-α (*P* < 0.01, *P* < 0.05).

### Tissue angiotensin II (Ang II) concentration

As depicted in Table 3, significant difference of Ang II concentration was not found in WKY with or without ACRS. The protein level of tissue Ang II was significantly increased in SHR model than WKY rats (*P* < 0.05). Treated with ACRS (140, 280 mg/kg) or captopril significantly lowed the protein level of tissue Ang II (*P* < 0.05).

### mRNA expression of collagen types I, III, transforming growth factor-β1 (TGF-β1) and angiotensin converting enzyme (ACE)

As depicted in Table 4, significant difference of collagen type I mRNA expression was not found in WKY with or without ACRS. The mRNA expression of collagen I was increased in SHRs than WKY rats obviously (*P* < 0.01). Treated with ACRS inhibited the mRNA over-expression of collagen type I (*P* < 0.01). Captopril decreased mRNA expression of collagen type I as well (*P* < 0.01). However, there was no significant difference of collagen type III mRNA expression among all the groups.

**Table 4:** Effects of active components of Radix Scrophulariae (ACRS) on mRNA expression of collagen types I and III, angiotensin converting enzyme (ACE) and transforming growth factor-β1 (TGF-β1) in spontaneously hypertensive rats (SHR) and Wistar–Kyoto rats (WKY) ($\bar{x}$ ± SE, n = 6)

Group	Collage I mRNA	Collage III mRNA	TGF-β1 mRNA	ACE mRNA
WKY control	1.03 ± 0.59	1.29 ± 0.86	0.62 ± 0.37	1.20 ± 0.59
WKY+ 140 mg/kg ACRS	1.47 ± 0.60	1.28 ± 0.85	0.85 ± 0.27	1.30 ± 0.42
SHR control	4.85 ± 2.60^††^	1.47 ± 0.68	1.58 ± 1.05^†^	5.51 ± 3.83^†^
SHR+ 40 mg/kg captopril	1.17 ± 0.28**	0.79 ± 0.34	0.77 ± 0.18*	1.35 ± 0.24*
SHR+ 70 mg/kg ACRS	1.39 ± 0.44**	1.32 ± 0.44	0.94 ± 0.22*	1.21 ± 0.23*
SHR+ 140 mg/kg ACRS	1.22 ± 0.51**	1.15 ± 0.51	0.60 ± 0.34*	1.25 ± 0.24*
SHR+ 280 mg/kg ACRS	1.22 ± 0.49**	1.08 ± 0.60	0.88 ± 0.18*	1.07 ± 0.20*

Values are expressed as the relative integrated intensity, and normalized to that of the GAPDH. Compared with WKY control group: ^††^ *P* < 0.01; compared with SHR control group: * *P* < 0.05; ** *P* < 0.01

As depicted in Table 4, significant difference of TGF-β1 and ACE mRNA expression was not found in WKY with or without ACRS. TGF-β1 and ACE mRNA expressions in SHRs were increased in SHRs than WKY rats obviously (*P* < 0.05). Treated with ACRS or captopril suppressing the mRNA over expression of TGF-β1 and ACE (*P* < 0.05).

### Mitogen-activated protein kinases (MAPKs) pathways

**Fig. 4:**
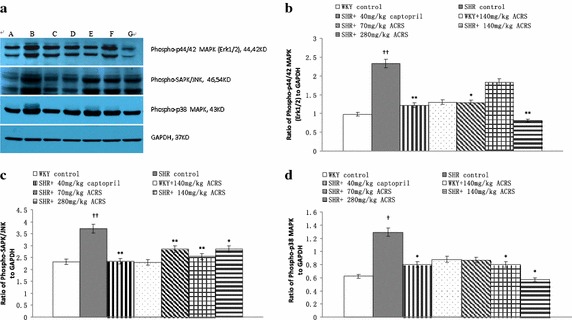
Effects of active components of Radix Scrophulariae (ACRS) on phospho-specific extracellular signa lregulated kinase (ERK 1/2), phospho-specific c-Jun N-terminal kinase (JNK) and phospho-p38 mitogen-activated protein kinases (p38 MAPK) in spontaneously hypertensive rats (SHR) and Wistar–Kyoto rats (WKY). **a** Representative western blot analysis is shown. **b** Densitometric evaluation of ERK 1/2 is shown. **c** Densitometric evaluation of JNK is shown. **d** Densitometric evaluation of p38 MAPK is shown. **A** WKY control; **B** WKY+ 140 mg/kg ACRS; **C** SHR control; **D** SHR+ 40 mg/kg captopril; **E** SHR+ 70 mg/kg ACRS; **F** SHR+ 140 mg/kg ACRS; **G** SHR +280 mg/kg ACRS. $\bar{x}$ ± SE, n = 6. Compared with WKY control group, ^††^ *P* < 0.01. Compared with SHR control group, *P < 0.05; **P < 0.01

As illustrated in Fig. 4, significant differences of the phosphorylation of extracellular signal regulated kinase (ERK1/2) Thr202-Tyr204, c-Jun N-terminal kinase (JNK/SAPK) Thr^183^–Tyr^185^ and p38 MAPK Thr^180^–Tyr^182^ were not found in WKY with or without ACRS. The phosphorylation of ERK1/2 Thr^202^–Tyr^204^, JNK Thr^183^–Tyr^185^ and p38 MAPK Thr^180^–Tyr^182^ was significantly increased in SHR model than that in WKY rats (*P* < 0.01), treated with ACRS or captopril attenuated the phosphorylation obviously (*P* < 0.01).

## Discussion

SHR is a useful experimental model of essential hypertension (Boluyt and Bing 2000; Bing et al. 2002). SHRs are characterized by the fact that they suffer from pre-hypertension during the first 6–8 weeks of their life and then develop many features of hypertensive end-organ damage: cardiac hypertrophy, cardiac failure and so on (Abbate et al. 2006). MAP is obviously increased in 16 weeks old (Shi et al. 2007). In agreement with the literature, we observed that MAP was much higher in SHR from 10 to 31 weeks of age than that in normotensive age-matched WKY. Administration of ACRS resulted in persistently lower MAP.

The increase in pressure overload was associated with a progressive left ventricular hypertrophy, as reflected by increased left ventricular mass to body mass ratio (Rysä et al. 2005). Cerutti et al. (2006) indicated that left ventricular mass index was highly and positively correlated to BP in the SHR. Other investigators also observed that SHR group had significantly greater HMI or/and LVMI than age-matched WKY group accompanied by blood pressure significantly increasing (Levick et al. 2006; Schultz et al. 2007; Shi et al. 2007). Consistently with these reports, in the present study, SHR exhibited an increased LVMI and HMI accompanied by increasing blood pressure, ACRS decreased blood pressure, LVMI and HMI.

Left ventricular hypertrophy (LVH) is one of the major risk factors underlying cardiovascular morbidity and mortality, which frequently observed in essential hypertension (Fortuño et al. 2003; Weber 2000). The development of LVH in hypertension is often regarded first as an adaptation to increased workload, while the transition to heart failure reflects the loss of efficacy of this process (Cerutti et al. 2006). Cingolani et al. reported that spontaneous hypertensive rats with compensated hypertrophy presented with a profile of compromised left ventricular diastolic function (Cingolani et al. 2003). In agreement with it, a progressive LVH was seen in SHR in our research, treatment with ACRS could obviously attenuate ventricular hypertrophy.

Histologically, the progression to heart failure is typically associated with increased fibrosis and disruption of normal cellular organization (Heyen et al. 2002). This leads initially to deleterious effects on diastolic function and subsequently to depressed systolic function due to interference with coordinated myocyte contraction. Hence, the amount of collagen in the myocardium seems to be a major determinant of the development of cardiac dysfunction in hypertension (Joseph et al. 2002). In an experimental setting, PVCA/LA (perivascular collagen area-to-lumen area ratio) and the myocardial interstitial collagen volume fraction (CVF) are indexes of perivascular and interstitial fibrosis, they were significantly increased in SHR compared with that in the WKY control (Shi et al. 2007). This is consistent well with our results that the interstitial and perivascular collagen in left ventricles was significantly increased in SHR compared with that in WKY rats at 31 weeks of age. And ACRS possessed the effect on maintaining a more advantageous mass, PVCA and CVF, which suggested that ACRS could attenuate the myocardium collagen accumulation in the SHR.

SHRs are characterized by sympathetic hyperactivity (Head 1989). There is evidence that the sympathetic nervous system (SNS) mediates hypertension-induced cardiac fibrosis and hypertrophy through α- and β-adrenergic receptors respectively. Perlini et al. (2005) further demonstrated that chemical sympathectomy in hypertensive rats prevented cardiac fibrosis. In the present study, an increased concentration of serum NE was detected in SHRs, and ACRS seemed to inhibit the sympathetic nerves to release NE. According to the results, we deduced that ACRS may slow down or even inhibit the process of cardiac fibrosis and hypertrophy by reducing sympathetic hyperactivity.

An important role for the renin-angiotensin system (RAS) in promoting hypertension and related end-organ damage is well established (Shigenaga et al. 2008). Ang II is the central role of the RAS. It is an octapeptide that induces multiple physiological responses in different cell types. In addition to its well-known vasoconstrictive effects, growing evidence supports the notion that Ang II may play a central role not only in hypertension but also in cardiovascular and renal diseases (Tamura et al. 2000). And the study of Varagic et al. (2008) provided evidence that Ang II mediated collagen deposition within the ventricles could be independent of pressure. In the present study, treatment with ACRS showed its importance in lowering hypertension as well as reducing Ang II concentration. The results indicated that the function of ACRS in antagonizing myocardial remodeling correlated well with its inhibition of the RAS.

Inflammation is also an important component in the development of hypertension-induced cardiac fibrosis (Kuwahara et al. 2004). TNF-α is among the most important inflammatory cytokines, plays a harmful role in progression of ventricular remodeling (Kassiri et al. 2005). It can depress cardiac function directly and indirectly by induction of nitric oxide synthase produced by macrophages, cardiac myocytes and other cells (Negrusz-Kawecka 2002). Our results showed that serum TNF-α concentration in SHRs was obviously higher than that in the WKY control rats, treatment with ACRS decreased the serum TNF-α concentration significantly, suggesting that antagonizing fibrosis and myocardial hypertrophy by ACRS may be at least partially correlated with its decreasing of the TNF-α concentration in SHRs.

Classically, genetic hypertension implies that abnormal gene expression in a normal environment in early life results in hypertension. Solid evidence indicates that Ang II directly stimulates left ventricular TGF-β1, and collagen gene expression independently of its hemodynamic effect (Kim et al. 1995; 1996). Collagen type I and type III are major fibrillar collagens involved in tissue repair (Sun and Weber 1996). An association of TGF-β1 over-expression with cardiac fibrosis has been reported in SHR (Shiota et al. 2003). Some studies showed that TGF-β1 could induce an increased collagen type I and type III synthesis in rats (Lijnen et al. 2000). And TGF-β1 was the major growth factor responsible for cardiac fibrosis (Berk et al. 2007). In our study, an increased accumulation of collagen type I and type III, the over-expression of collagen type I mRNA and TGF-β1 mRNA in SHR were significantly suppressed by ACRS as well as captopril. The results indicated that the function of ACRS in inhibiting cardiac fibrosis might be mediated by its inhibition of TGF-β1 and collagen type I mRNA over-expression.

A number of specific candidate genes are implicated in the pathogenesis of hypertension in SHR. These include components of the renin-angiotensin system such as ACE (Zhang et al. 1996). Not surprisingly, the beneficial action of ACE inhibitors on target organ damage in the heart has been shown in a variety of clinical settings (Enseleit et al. 2001). Chronic treatments with ACRS at doses of 70, 140, and 280 mg/kg per day for 21 weeks were all effective in reducing the mRNA expression of ACE in SHR. It indicated that the inhibitory effects of ACRS on ACE might be one of the contributing factors in restraining ventricular remodeling in SHR.

Many studies have revealed an increase of MAPK (ERKs, p38, and JNKs) during hypertension (Takeishi et al. 2001; Aoyagi and Izumo 2001). Growing evidence suggests that modulation of the complex network of MAPKs cascades should be a rewarding approach to the treatment of ventricular hypertrophy and HF (Luedde et al. 2006). In this study, we surveyed tyrosine/threonine phosphorylation of the major MAP kinases and the data showed that the activity of ERK1/2 was significantly increased in SHR compared with normotensive control. Similarly, p38 MAPK and JNK/SAPK phosphorylation activities were increased in SHRs compared with normotensive control. And the increased phosphorylation activations of ERK1/2, JNK and p38 MAPK in the SHR were decreased by treatment with ACRS, which showed that ACRS played a beneficial role in ventricular remodeling may through alteration of MAP kinases signaling.

## Conclusion

Treatment of ACRS for 21 weeks could beneficially reduce blood pressure, lower myocardium hypertrophy, reduce amount of interstitial and perivascular collagen, lower the accumulation of collagen types I and III. The mechanism may be related to its restraining the hyperactivity of SNS and RAS, suppressing the over-expression of ACE, TGF-β1 and collagen type I mRNA over-expression and the activation of signaling pathways of ERK 1/2, JNK and p38 MAPK.

## Methods

### Drugs and reagents

The preparation of ACRS was described as previous study (Huang et al. 2012). Four main peaks shown in the HPLC chromatograms of ACRS were Harpagide, Harpagoside, angoroside C and cinnamic acid, account for 18.7, 13.4, 14.6, 5.7 % respectively. The rest main constituents were polysaccharides in ACRS.

Captopril tablets were suspended in distilled water before use (Jiangsu Huanghe River Pharmaceutical Co., Ltd. China. Lot Number: 090727).

### Experimental schedule

Male SHR and control Wistar–Kyoto rats (WKY) (Shanghai Slac laboratory animal Co., Ltd. China) were housed in Laboratory Animal Center with a 12 h light/dark cycle and they had free access to chow and water. The temperature was at 22–24 °C and the humidity was at 40 ± 5 %. Sodium pentobarbital anesthesia was used in the surgery to minimize suffering.

All performance followed the Guide for the Care and Use of Laboratory Animals (1996, published by National Academy Press, 2101 Constitution Ave. NW, Washington, DC 20055, USA).

After 10 days of acclimatization to this facility, blood pressure was test in WKY rats and SHRs by tail cuff. And then SHRs were randomized divided into four groups: SHR model, SHR with ACRS (70,140, 280 mg/kg) and captopril (40 mg/kg), n = 8. WKY rats were randomized to two groups: WKY with ACRS (140 mg/kg) and WKY control, n = 8.

Then animals in every group were allowed free access to the same administered. Animals were treated with captopril or ACRS by gavage at corresponding doses daily, continued for 21 weeks. MAP was recorded every 3 weeks. Body mass (BM) was monitored weekly.

### Calculation of left ventricular mass index

The animals were anesthetized with urethane (1.0 g/kg) intraperitoneally and blood sample was collected. Then the hearts were isolated and weighed. The LVMI and HMI were calculated by the left ventricular mass to the BM ratios and the heart mass to the BM (Gao et al. 2010; Huang and Chen 2013). The lower part of the left ventricular was frozen at −70 °C before assaying. The upper part of the left ventricular was immersed in 10 % formaldehyde.

### Histological analysis

Myocardial segments from the upper part were imbedded in the paraffin, sectioned (5 mm) and stained with Sirius red. Then CVF and PVCA were calculated. Accumulation of types I and III collagen in the interstitial and perivascular space of the left ventricle was assessed by polarized light microscopy (Gao et al. 2010; Huang and Chen 2013).

The microscope (Olympus BX51, Japan) was used to photograph each sample slice (400× magnification). The image-Pro + 6.3 analyzing software (Media Cybernetics, Bethesda, MD, USA) was used to analyze all photos.

### ELISA analysis

Serum level of NE was determined by enzyme-linked immunosorbent assay (ELISA) kit (Shanghai Xitang Institute of Bioengineering, Shanghai, China).

### Radioimmunoassay determination

Radioimmunoassay was used to assay TNF-α concentration in serum and Ang II concentration in tissue. The supernatant of homogenized tissue was obtained by centrifugalization (4 °C, 1780*g*, 15 min) TNF-α and Ang II concentrations were analyzed with Iodine [125I] TNF-α kit and Iodine [125I] Ang II kit (Product of Beijing North Institute of Biological Technology, China). A Coomassie Brilliant Blue Kit (Nanjing Jiancheng Institute of Bioengineering, China) was applied to measure protein concentrations of the homogenate supernatants. Serum TNF-α was expressed as concentration per milliliter serum sample. Tissue Ang II was expressed as per milligram protein.

### Real-time RT-PCR determination

Analysis of mRNA expression levels for collagen types I, III, TGF-β1 and ACE were performed with primers designed to detect rat gene products: Collagen type I used primers, FWD: 5′-CCTGCCGATGTCGCTATCC-3′, and REV: 5′-TTGCCTTCGCCCCTGAG-3′; Collagen type III used primers, FWD: 5′-GCCTCCCAGAACATTACATACC-3′, and REV: 5′-CTGTCTTGCTCCATTCACCAG -3′; TGF-β1 used primers, FWD: 5′-TGGCGTTACCTTGGTAACC-3′, and REV: 5′-GGTGTTGAGCCCTTTCCAG-3′; ACE used primers, FWD: 5′-ATGAGGCTATTGGAGATGTTTTG-3′, and REV: 5′-TCCTTGGTGATGCTTCCGT-3′; GAPDH used primers, FWD: 5′-TGGCATGGACTGTGGTCATG-3′, and REV: 5′-TGGGTGTGAACCACGAGAAA-3′.

Real-time RT-PCR and datum analysis were carried out with realplex 7500 (Applied Biosysems, USA).

All data obtained with collagen types I and III, TGF-β1, ACE primers were normalized to the GAPDH primers, namely the relative integrated intensity was taken.

### Western blotting

Western blots were measured as previously described (Huang and Chen 2013). Briefly, active and total form of ERK1/2, JNK and p38 MAPK accumulation were detected by Western blot analysis with using the following antigenes: phospho-P44/42MAPK (ERK 1/2) Thr^202^–Tyr^204^ (1:2000), phospho-SAPK/JNK Thr^183^–Tyr^185^ (1:1000), phospho-p38 MAPK (Thr^180^–Tyr^182^) (1:1000) (Cell Signaling Technology, Beverly, MA, USA), anti-GAPDH (Abmart, Shanghai, China), and a goat anti-rabbit horseradish peroxidase-conjugated secondary antibody (1:5000 dilution, Santa Cruz). Bands were visualized by means of enhanced chemiluminescence. Then NIH Image J program was used to quantify the results after scanning.

### Statistical analysis

Results were expressed as mean ± SD. For evaluating the difference between two groups, the Student–Newman–Keuls test and one-way analysis were used. Statistical analysis was performed by SPSS 13.0 software for multiple comparisons. *(^#^), **(^##^), and ***(^###^) denote P < 0.05, <0.01 and <0.001, respectively.

## Abbreviations

ACRS: active components from Radix Scrophulariae
VR: ventricular remodeling
SHR: spontaneously hypertensive rat
WKY: Wistar–Kyoto rats
Ang II: angiotensin II
TNF-α: tumor necrosis factor-α
TGF-β1: transforming growth factor-β1
ACE: angiotensin converting enzyme
ERK1/2: extracellular signal regulated kinase ½
JNK: c-Jun N-terminal kinase
MAPK: mitogen-activated protein kinases
HF: heart failure
MAP: mean arterial pressure
LVMI: left ventricular mass index
HMI: heart mass index
CVF: myocardial interstitial collagen volume fraction
PVCA: perivascular collagen area to vessel lumen area ratio
NE: norepinephrine
